# Electrochemotherapy for cutaneous tumors combined with tumor irradiation before or after treatment: feasibility, safety, and effectiveness

**DOI:** 10.2478/raon-2026-0051

**Published:** 2026-09-07

**Authors:** Giulia Bertino, Marta Minuti, Julie Gehl, Erika Kis, Hadrian Nassabi, Michele Pio Grieco, Giuseppe Riva, Matteo Mascherini, Francesca De Terlizzi, Maja Cemazar, Gregor Sersa

**Affiliations:** 1 Department of Otolaryngology Head and Neck Surgery, University of Pavia, IRCCS Policlinico San Matteo Foundation, Pavia, Italy; 2 Department of Clinical Oncology and Palliative Care, Zealand University Hospital, Roskilde, Denmark; 3 Department of Clinical Medicine, Faculty of Health and Medical Sciences, University of Copenhagen, Copenhagen, Denmark; 4 Department of Dermatology and Allergology, Albert Szent-Györgyi Medical School, Albert Szent-Györgyi Clinical Centre, University of Szeged, Szeged, Hungary; 5 Department of Dermatology and Plastic Surgery, SRH- Waldklinikum, Gera, Germany; 6 UOC Chirurgia Plastica e Skin Cancer Unit, IRCCS CROB Centro Riferimento Oncologico Basilicata, Rionero in Vulture (PZ), Italy; 7 Division of Otorhinolaryngology, Department of Surgical Sciences, AOU Città della Salute e della Scienza of Turin, Torino, Italy; 8 Department of Surgery, IRCCS AOM Liguria, Genoa, Italy; 9 IGEA Biophysics Lab. Carpi, Modena, Italy; 10 Department of Experimental Oncology, Institute of Oncology Ljubljana, Slovenia

**Keywords:** cutaneous tumors, electrochemotherapy, tumor irradiation, neoadjuvant treatment, salvage therapy, tumor control

## Abstract

**Background:**

Electrochemotherapy (ECT) can be performed on tumors in patients that precedes tumor irradiation or on recurrent tumors after tumor irradiation. In this study we evaluated the feasibility, safety and effectiveness of the combined ECT and tumor irradiation (radiotherapy, RT) on different tumors in the head and neck region.

**Patients and methods:**

For the present cohort analysis, patients were selected from the Insp-ECT database, based on the following criteria: presence of head and neck lesions of any histological origin, treatment with both ECT and RT in any sequence, and a maximum interval of 12 months between the two treatments. The patients were treated with ECT followed by RT (ECT → RT) or RT followed by ECT (RT → ECT), in both cases because the first treatment resulted in incomplete response or subsequent relapse. In the case of ECT → RT the meantime interval was 5.7 months and in the case of RT → ECT 7.6 months. The minimal time interval between the treatments was 1 month.

**Results:**

Treatment with ECT with bleomycin was feasible in all patients, treated either before or after RT. Included were 37 patients with the head and neck lesions. The combined treatment was performed predominantly on older population of patients with the age range from 50 to 96 years, median over 80 years. Importantly, in the group ECT → RT the major reason for RT was partial response (67%) after ECT. While, in the RT → ECT group the major reason for subsequent ECT was 58% of stable or progressive disease (failure to treatment). In both groups local symptoms decreased, like ulceration, odour and suppuration of tumors. In the ECT → RT group, the short-term response after 2 months was high, with 89% objective responses. In contrast, the RT → ECT group had a significantly lower rate, with 58% objective responses. At 24-month progression free survival (PFS) was 68% in the ECT → RT group and 20% in the RT → ECT group (p = 0.0101). The overall progression free survival Kaplan-Meier curves also differed significantly (log-rank p = 0.0232).

**Conclusions:**

The combined treatment of ECT and RT is feasible, safe, and effective in elderly patients. The findings suggest that ECT may have particular value as a neoadjuvant treatment before RT, especially for bulky, ulcerated, or symptomatic lesions in this population.

## Introduction

Electrochemotherapy (ECT) is an effective local ablative therapy that utilizes application of electric pulses for efficient drug delivery into tumor cells.^1^ The local potentiation of the drug, either bleomycin or cisplatin into cells provides effective tumor cell cytotoxicity. ECT has been proven in many clinical studies to be effective in local control of cutaneous as well as some deep-seated tumors.^2-12^

The Insp-ECT consortium collects clinical data on the treatment of cutaneous tumors with ECT from 48 cancer centers across Europe. The comprehensive report from this consortium reported an 85% objective response rate of the treated tumors across various tumor histotypes.^3^ This response rate depends to some degree on tumor type, with melanoma being the least responsive, and on the tumor size, larger than 2 cm in diameter are less responsive. Another clinical factor influencing treatment effectiveness is previous treatment, such as chemotherapy or radiotherapy. The clinical data indicate that treatment naïve tumors respond better than previously irradiated tumors.^3^

Preclinical data indicate a radiosensitizing effect of ECT with bleomycin and cisplatin.^13-18^ The systematic review by Ferioli *et al*.^19^, comprehensively summarizes all the preclinical studies. The studies explored the best treatment regimen, ECT before or during the beginning of the radiation regimen resulted in pronounced tumor response. There are already some clinical reports, mainly case series, indicating that combining ECT with tumor irradiation is feasible and effective with local mild minimal side effects.^20–23^

However, if the interval between ECT and tumor irradiation extends to weeks or months, the radiosensitizing effect, which was demonstrated in preclinical studies and some clinical reports, is likely no longer present. No studies have yet examined the effectiveness of combining ECT before or after RT, with the interval between them at least one month. Therefore, the aim of this study was to evaluate the feasibility, safety, and effectiveness of combined ECT before or after RT in different tumor types, with head and neck squamous cell carcinoma (SCC) as the predominant type. In both scenarios, patients received the second therapy because of incomplete response or subsequent relapse.

## Patients and methods

Data for this analysis were extracted from the Insp-ECT database, which includes contributions from 48 European centers reporting their experience with ECT. All data on patients treated with ECT are prospectively entered into a centralized online registry (https://Insp-ECT.eu) in anonymized form.

The Insp-ECT Registry contains comprehensive information on patient demographics, Eastern Cooperative Oncology Group (ECOG) performance status, disease stage, prior and concomitant oncologic treatments, as well as the location, number, size, and characteristics of cutaneous metastases. In addition, detailed data on ECT procedures (including anesthesia type and treatment parameters), treatment-related toxicities and adverse events, clinical response, pain assessment, and quality of life are systematically recorded.^3,24^

All patients provided written informed consent prior to inclusion. Data was entered prospectively by each participating center and updated over time. Each institution obtained approval from its local Ethics Committee and relevant data protection authorities. Participation in the registry required a formal signed agreement. The study was conducted in accordance with the principles of the Declaration of Helsinki.

For the present cohort analysis, patients were selected based on the following criteria: presence of head and neck lesions of any histological origin, treatment with both ECT and RT in any sequence, and a maximum interval of 12 months between the two treatments. Additional data were collected regarding treatment sequencing (ECT after RT or RT after ECT), as well as RT modality and delivered dose. Eight centers contributed to the patients’ data collection: Pavia (IT), Ljubljana (SLO), Copenhagen (DK), Mainz (DE), Szeged (HU), Rionero in Vulture (IT), Torino (IT), Genova (IT).

### ECT treatment

ECT was administered according to the most recent European Standard Operating Procedures (ESOPE) guidelines, as previously described.^25,26^ Each participating center was responsible for defining the therapeutic strategy and making clinical decisions for individual patients, including the choice of anesthesia (general or local) based on clinical indication and patient preference.

Bleomycin was administered either intratumorally at a dose of 1000 IU/mL/cm^3^ or intravenously at 15,000 IU/m^2^. Electroporation was performed using the Cliniporator device (IGEA, Carpi, Italy), delivering eight pulses of 100 μs at an electric field strength of 1 kV/cm (adjusted to electrode spacing; for example, 400 V for a 4 mm electrode gap). Electrode selection was performed in accordance with standard operating procedures.^25,26^

### Radiotherapy treatment

External beam radiation therapy (RT) was administered for the treatment of superficial cutaneous metastatic lesions in the head and neck region.^27,28^ Treatment technique was selected at the discretion of each participating center based on lesion characteristics, anatomical location, and institutional practice. Photon-based approaches included intensity-modulated radiotherapy (IMRT) and image-guided radiotherapy (IGRT), allowing for improved dose conformity and setup accuracy, respectively.^29^ In cases of superficially located lesions, electron beam radiotherapy was employed to exploit its limited penetration depth and rapid dose fall-off beyond the target volume, thereby minimizing exposure to underlying normal tissues. Details regarding the specific technique and total prescribed dose were collected and included in the analysis.

### Response evaluation

Local response following ECT and/or RT was assessed 45–90 days after completion of the last treatment, according to modified Response Evaluation Criteria in Solid Tumors (RECIST).^30^ Briefly, complete response (CR) was defined as the disappearance of the target lesion. Partial response (PR) was defined as a ≥30% reduction in the diameter of the target lesion. Progressive disease (PD) was defined as a ≥20% increase in lesion diameter, while stable disease (SD) was defined as neither sufficient shrinkage to qualify for PR nor sufficient increase to qualify for PD.

Up to seven cutaneous metastases per patient (including the largest lesion) were selected and recorded as target lesions.

### Statistical analysis

Continuous variables were summarized using median values and ranges, while categorical variables were reported as absolute frequencies and percentages. Group comparisons for continuous variables were performed using a two-tailed heteroscedastic Student’s t-test, and categorical variables were compared using the chi-square test.

Tumor control was evaluated in terms of progression-free survival (PFS), defined as the time from the first treatment (either RT or ECT) to disease progression, relapses, or last follow-up, whichever occurred first. Survival curves were estimated using the Kaplan–Meier method. One-year and 2-years local PFS rates and corresponding 95% confidence intervals (95% CI) were calculated. Differences between survival curves were assessed using the Mantel–Haenszel (log-rank) test.

All statistical analyses were performed using NCSS version 9 (NCSS, LLC, Kaysville, UT, USA). A p-value < 0.05 was considered statistically significant.

## Results

### Patient characteristics and treatment data

In the analysis, 37 patients with lesions in the head and neck region were included. All patients were treated at 8 European centers within the Insp-ECT group. All patients received both ECT and RT within 12 months and were listed into two groups: the “ECT → RT” group received ECT as the first treatment and RT as the subsequent treatment; the “RT → ECT” group received RT as the first treatment and ECT as the subsequent treatment.

Both groups were well matched in demographics, including number of patients, age, and nodule size (Table 1). The predominant tumor types in both groups were squamous cell carcinoma (SCC) and basal cell carcinoma (BCC), along with melanoma, breast cancer, soft tissue sarcoma, and others (Table 1). Tumors were located in the head and neck region. The nodule size was relatively large in both groups, with a mean diameter exceeding 30 mm and ranging from 2 mm to 170 mm in the largest diameter.

**TABLE 1.** Characteristics of the 2 groups of patients treated with combined treatment with ECT and RT

	ECT → RT (N = 18)			RT → ECT (N = 19)			P
	**TYPE**	**N**	**%**	**TYPE**	**N**	**%**	**Value**
DIAGNOSIS*	MM	0	0%	MM	1	5%	0.8896
	BCC	7	40%	BCC	5	26%	
	MA	1	5%	MA	2	11%	
	SCC	9	50%	SCC	9	48%	
	SAR	1	5%	SAR	1	5%	
	OTH	0	0%	OTH	1	5%	
**T-STAGE**	T1	3	17%	T1	6	32%	0.5381
	T2	10	56%	T2	6	32%	
	T3	1	5%	T3	4	20%	
	T4	3	17%	T4	3	16%	
	TX	1	5%	TX	0	0%	
	**MEAN**	**ST.DEV**.	**MEDIAN (RANGE)**	**MEAN**	**ST.DEV**.	**MEDIAN (RANGE)**	
**AGE**	80	10	83 (59-94)	81	13	87 (50-96)	0.7102
**NODULES’ SIZE (mm)**	36	25	30 (14-130)	40	41	30 (2-170)	0.6513
**MONTHS BETWEEN TREATMENTS**	5.7	3.6	5.3 (1-12)	7.6	4.1	8 (1-12)	0.1587

1 BCC = basal cell carcinoma, MA = breast cancer; MM = melanoma; OTH = other; SAR = soft tissue sarcoma; SCC = squamous cell carcinoma

1 In ECT → RT group OTH was neoplasia of the right parotid region, in RT → ECT group OTH was Metastatic Transitional Cell Carcinoma of maxillary sinus.

The interval between the ECT → RT and RT → ECT groups were not significantly different (p = 0.1587), with mean times of 5.7 and 7.6 months, respectively (Table 1). The minimum interval was set at 1 month, which is important because we wanted to exclude patients in whom the radiosensitizing effect of ECT could be significant, due to radio sensitizing effect of bleomycin. After 1 month, bleomycin is expected to have been eluted from the ECT-treated tumors, as the anti-tumor effect of ECT should have been fully exerted by then.

The predominant type of electrodes used in ECT were linear and hexagonal array electrodes in both treatment groups. Predominant type of drug administration was intravenous, although relatively high percentage of patients in both groups received intratumoral bleomycin administration (22% and 32%, respectively) (Table 2). The dosage of bleomycin administered was according to the standard operating procedures, for either intravenous or intratumoral administration. The prescribed radiation dose did not differ significantly between the two groups, with a mean dose of 49 ± 13 Gy in the ECT → RT group and 41 ± 14 Gy in the RT → ECT group (p = 0.1853). Photon-based radiotherapy was the predominant treatment modality in both cohorts (Table 2).

**TABLE 2.** Data on treatments (RT and ECT) in the two groups of patients

	ECT → RT (N = 18)			RT → ECT (N = 19)			P
	**TYPE**	**N**	**%**		**N**	**%**	**Value**
**ELECTRODE**	plate	0	0%	plate	2	11%	0.3955
	linear	8	44%	linear	8	42%	
	hexagonal	9	50%	hexagonal	9	47%	
	multiple	1	6%	multiple	0	0%	
**DRUG**	I.V.	14	78%	I.V.	13	68%	0.7140
**ADMINISTERED**	I.T.	4	22%	I.T.	6	32%	
**IRRADIATION**	photons	14	78%	photons	15	79%	1.000
	electrons	4	22%	electrons	4	21%	

The reasons for the second treatment differed between the two groups of patients. In the group of ECT → RT the major reason was partial response to ECT; therefore, tumor irradiation was performed in 67% of patients. In contrast, in the RT → ECT group of patients ECT was performed after failure of response to RT in 58% of cases (Table 3). The second treatment in both groups of patients was rarely performed because of the relapse after previous treatment. Considering this difference in the approaches the reasons also differed statistically significantly (p = 0.0423).

**TABLE 3.** Reasons for the second treatment in the two groups of patients (p = 0.0423)

REASON FOR 2ND TREATMENT	After PR of previous treatment	After SD or PD of previous treatment	Relapse after CR of previous treatment
**ECT → RT (N=18)**	12	4	2
Reason for RT	67%	22%	11%
**RT → ECT (N=19)**	5	11	3
Reason for ECT	26%	58%	16%

### Feasibility and safety

In both groups of patients, the combined treatment either ECT before or after the tumor irradiation was feasible, according to the current standard operating procedures.

**FIGURE 1.**
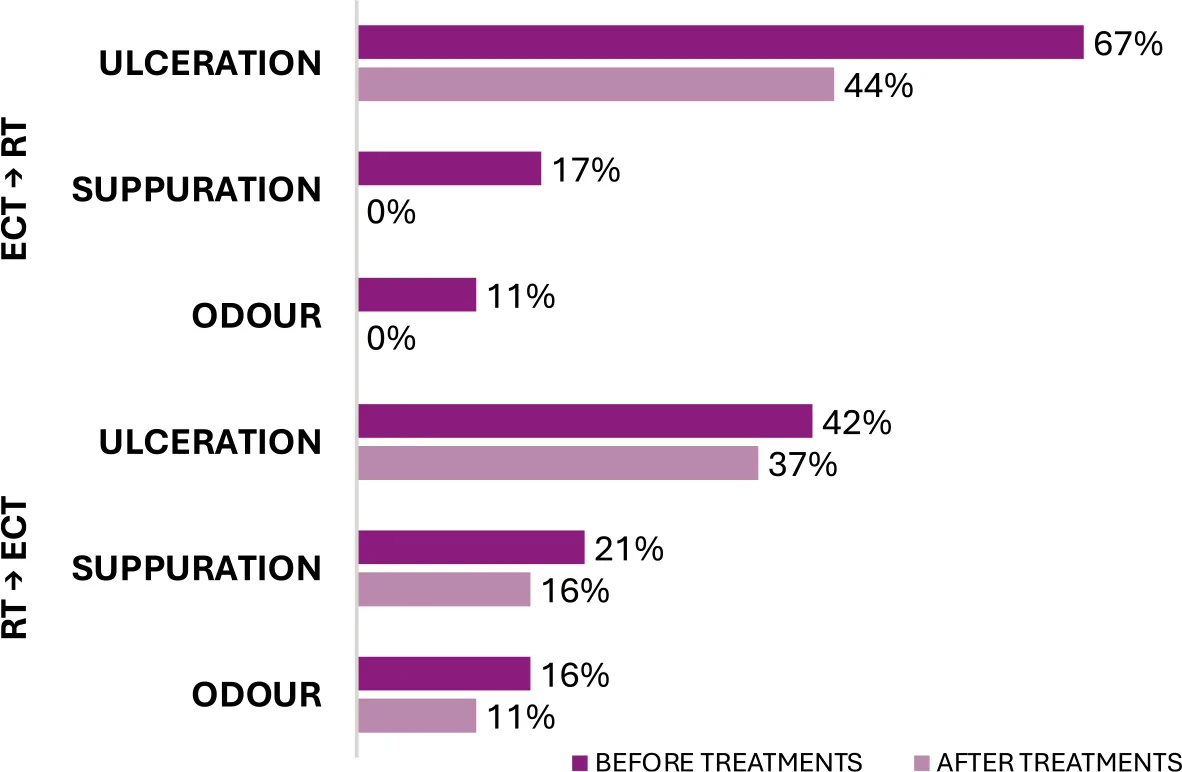
Incidence of most common local symptoms in the two groups before and after the treatments.

The treatment was also safe. In both groups the local symptoms, incidence and gravity decreased. Ulceration decreased from 67% to 44% in the ECT → RT group and from 42% to 37% in RT → ECT group. Odour decreased from 11% to 0% in the ECT → RT group and from 16% to 11% in the RT → ECT group. Suppuration decreased from 17% to 0% in the ECT → RT group and from 21% to 16% in the RT → ECT group (Figure 1).

### Short-term response

**FIGURE 2.**
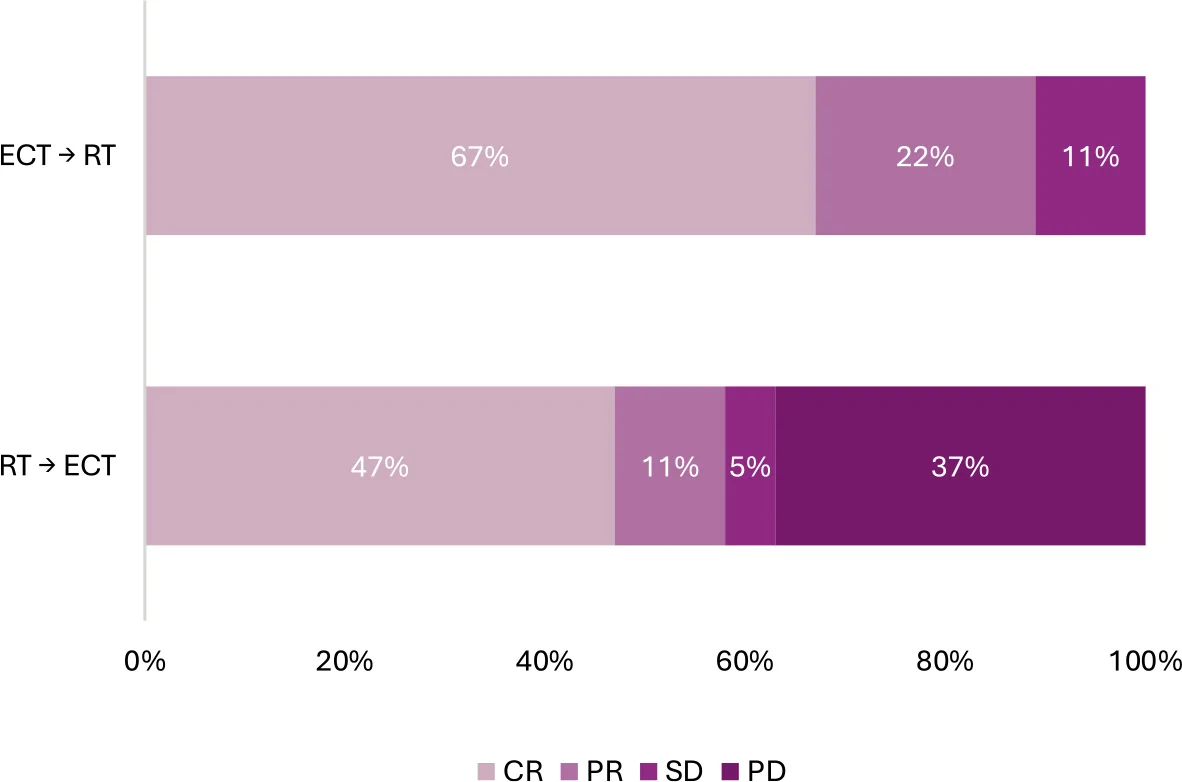
Distribution of short-term response in the 2 groups after the sequence of both treatments. CR = complete response; PR = partial response; SD = stable disease; PD = progressive disease

Short term response was assessed at 2 months of follow-up after treatment. The distribution of response categories differed significantly between the two groups (p = 0.0383), with a higher proportion of CR and PR and no PD in the ECT → RT group (Table 4). No progressive disease was observed in the ECT → RT group, whereas PD occurred in 37% of patients in the RT → ECT group. Figure 2 illustrates the higher response rate observed with the ECT → RT sequence.

**TABLE 4.** Short term response in the 2 groups after the sequence of both treatments

	ECT → RT		RT → ECT		P VALUE
	**N**	**%**	**N**	**%**	
**CR**	12	67%	9	47%	0.0383
**PR**	4	22%	2	11%	
**SD**	2	11%	1	5%	
**PD**	0	0%	7	37%	

1 CR = complete response; PR = partial response; SD = stable disease; PD = progressive disease

### Long term response

Patients in the ECT → RT group were followed for a mean time of 23 ± 21 months (median 14, range 4-79 months). At last follow-up 7 (39%) had no evidence of disease, 5 (28%) were alive with disease, and 6 (33%) died. In the RT → ECT group the mean follow-up time was 19 ± 13 months (median 17, range 3-53). At last follow-up 7 (37%) had no evidence of disease, 6 (32%) were alive with disease and 6 (32%) died.

During follow-up period, 4 recurrences/progressions were observed in the ECT → RT group (22%) and 12 were observed in the RT → ECT group (63%), significantly higher (p = 0.0201) than the former group.

**FIGURE 3.**
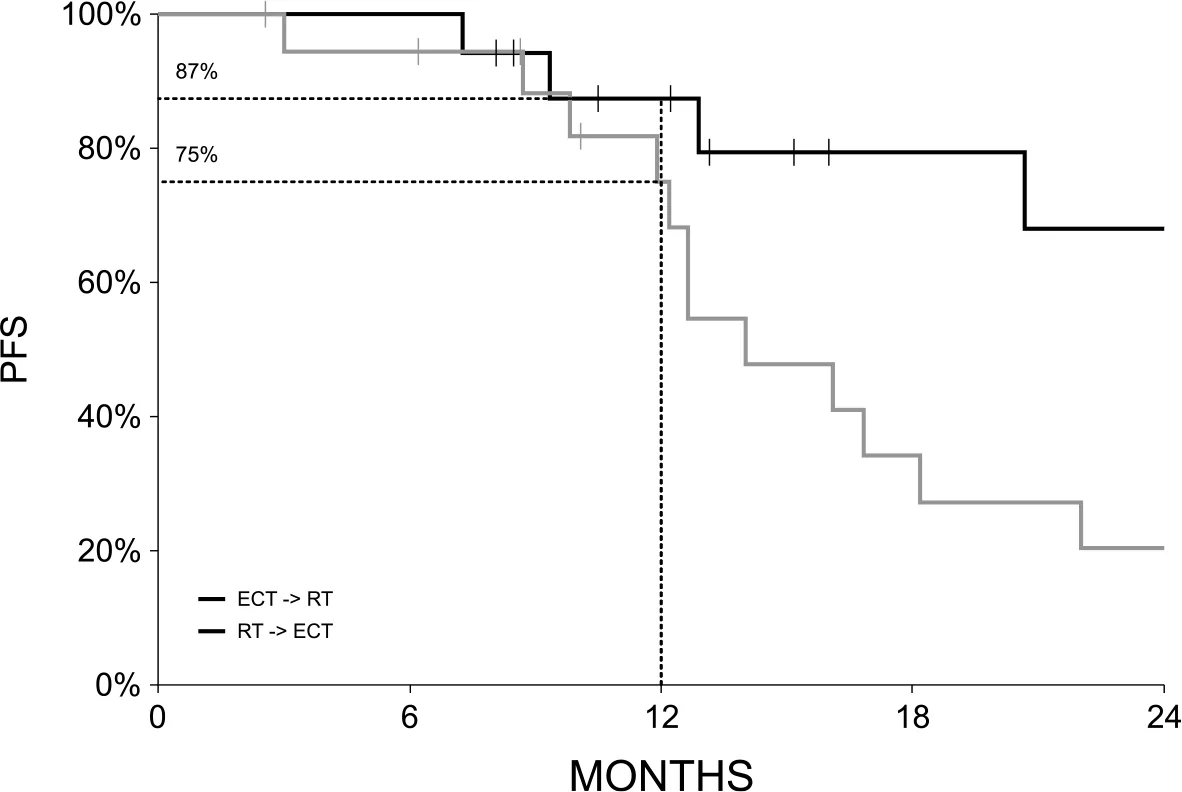
Progression free survival (PFS) Kaplan-Meier curves.

Progression free survival curves are presented in Figure 3. One-year progression free survival was 87% [C.I. 71%-100%] in the ECT → RT group and 75% [C.I. 54%-96%] in the RT → ECT group (p = 0.3819).

The recurrent tumors, which dominated in RT → ECT group, occurred after the first year and resulted in significant differences in progression free survival of the patients at 24 months as reported in Figure 3. In the ECT → RT group was 68% [C.I. 41%-95%], in the RT → ECT group it was 20% [C.I. 0%-41%] (p = 0.0101). This resulted in also significant difference (p = 0.0232) in the progression free Kaplan-Meier survival curves due to the higher progression free survival of the ECT → RT group.

## Discussion

This multicenter retrospective cohort analysis demonstrates that the combination of ECT and RT, administered either before or after tumor irradiation, is feasible, safe, and clinically effective in elderly patients with head and neck cutaneous malignancies. The study specifically evaluated treatment sequences separated by at least one month, thereby minimizing the likelihood that the observed effects were attributable to the known short-term radiosensitizing properties of bleomycin-based ECT.^13,14,22^ Despite the heterogeneous tumor histologies and treatment techniques, both strategies achieved meaningful local tumor control and symptom palliation.

The present results confirm previous clinical observations suggesting that ECT can be safely integrated with RT in the management of difficult-to-treat cutaneous tumors. While earlier reports mainly focused on concomitant or closely sequenced treatments designed to exploit radio-sensitization^13,14,22^, our study explored a different clinical scenario in which ECT and RT were combined over longer intervals. This distinction is clinically relevant because it reflects real-world treatment pathways in frail or elderly patients who often require staged therapeutic approaches. The mean interval between treatments was 5.7 months in the ECT → RT group and 7.6 months in the RT → ECT group, well beyond the expected persistence of bleomycin within treated tissues. Therefore, the improved outcomes observed in the ECT → RT cohort are unlikely to be explained solely by direct radiosensitization.

An important finding of this study is the significantly better short-term response observed when ECT preceded RT. The ECT → RT group achieved an objective response rate of 89%, compared with 58% of patients treated with RT followed by ECT. Moreover, no progressive disease was observed in the ECT → RT cohort, whereas more than one-third of patients in the RT → ECT group experienced disease progression despite salvage ECT. These findings suggest that ECT administered neoadjuvantly before irradiation may improve local disease conditions and enhance the effectiveness of subsequent RT. It is worth mentioning that ECT as the first treatment resulted in 67% of PR and that RT was performed on down-sized tumors. Thus, tumor downsizing achieved by ECT may reduce hypoxic tumor burden, and improve dose distribution, particularly in bulky and ulcerated lesions frequently encountered in the head and neck region. On the contrary, ECT after RT may be less effective because of tumor vessels damage due to RT, and consequently lower uptake and distribution of bleomycin after intravenous administration.

The clinical role of ECT differed substantially between the two treatment groups. Although the tumor size at the time of the first treatment did not differ between the groups, in the ECT → RT cohort, ECT was predominantly used as a neoadjuvant treatment prior to RT, mainly to reduce tumor volume and improve local tumor conditions before RT; in fact, in the ECT → RT cohort, 67% of patients underwent RT after achieving a partial response to ECT, and the final objective response rate after completion of the combined treatment was 89%. In contrast, ECT in the RT → ECT group was mainly used as salvage therapy after inadequate response or failure of irradiation (SD or PD after RT), resulting in only 58% of OR after the ECT. The sequence of treatments likely contributed to the poorer outcomes observed in the RT → ECT cohort, since previously irradiated tumors are known to be more resistant to subsequent local therapies because of fibrosis, vascular damage, hypoxia, and impaired tissue healing. Previous analyses from the Insp-ECT consortium have similarly shown that treatment-naïve lesions respond better to ECT than previously irradiated tumors.^3^ However, further studies on bigger cohorts of patients are needed to confirm that ECT resulted in better response than RT as the first treatment.

In addition to tumor response, both treatment strategies provided clinically meaningful symptom control. Clinically measured outcomes; reductions in ulceration, suppuration, and malodor were observed in both groups after treatment. These benefits are particularly relevant in elderly patients with advanced head and neck tumors, where local symptoms substantially impair quality of life, nutrition, communication, and social functioning. Therefore, even in patients with limited curative options, ECT combined with RT may provide important palliative benefits with acceptable tolerability.

Long-term tumor control also favored the ECT → RT sequence. Although both groups demonstrated encouraging progression-free survival (PFS), the ECT → RT group had significantly fewer recurrences and superior Kaplan–Meier PFS curves. One-year PFS reached 87% in the ECT → RT group compared with 75% in the RT → ECT group, with significantly fewer progression events overall. These findings further support the hypothesis that ECT may have particular value when integrated earlier in the local treatment strategy rather than being reserved exclusively as salvage therapy after RT failure.

The study population consisted predominantly of elderly patients, with median ages above 80 years in both cohorts. This aspect deserves particular attention because older patients are frequently poor candidates for extensive surgery or aggressive systemic therapies due to frailty and comorbidities. The good tolerability and feasibility observed in this study indicate that combined ECT and RT may represent a valuable therapeutic option for this challenging patient population. Importantly, treatment was successfully delivered across multiple European centers using standard operating procedures, supporting the reproducibility and general applicability of the approach.

Several limitations of this study should be acknowledged. First, the retrospective nature of the analysis and the relatively small sample size limit the strength of definitive conclusions. Second, the patient population was heterogeneous with respect to tumor histology, stage, RT techniques, and radiation doses. Third, the interval between ECT and RT varied considerably among patients and among groups, even if not significantly, which may have influenced treatment outcomes. Finally, because treatment allocation was not randomized, differences between groups may partially reflect underlying differences in disease biology and treatment intent, particularly the higher proportion of salvage cases in the RT → ECT cohort. This paper does not address the question whether primary treatment with ECT is more or less efficient than RT, since the database does not contain data on response rates of all patients treated with RT, only those that received ECT in addition to RT.

Nevertheless, this study provides clinically relevant evidence supporting the integration of ECT and RT in the treatment of head and neck cutaneous tumors. The findings suggest that ECT may have particular value as a neoadjuvant treatment before RT, especially for bulky, ulcerated, or symptomatic lesions in elderly patients. Future prospective studies should further investigate optimal sequencing, timing, and patient selection criteria for combined ECT and RT protocols. In particular, the promising results observed in the ECT → RT group support the hypothesis that ECT could be intentionally incorporated into future treatment strategies as a neoadjuvant approach to improve RT effectiveness and local tumor control.
